# Kerosene for Heat in Vulcanizing, &c.

**Published:** 1865-01

**Authors:** T. B. Welch


### KEROSENE FOR HEAT IN VULCANIZING, &C.

BY T. B. WELCH.

For the last six months I have been using Kerosene for Vulcanizing, &c. Thinking that a description of my apparatus may be of service, I give it to the profession.

It consists of an ordinary size kerosene hanging lamp, with a good size burner; a sheet iron sheath for holding the vulcanizer, with a sheet iron chimney attached.

This sheath is one and a half inches in diameter larger than the boiler, and stands upon three legs supporting it over the cone of the lamp, the hole in the bottom of the sheath, just fitting the cone around the blaze. The bottom of the vulcanizer is brought to within one inch of the lamp cone. The sheath is of two parts: the upper two-thirds made to fit on to other third; put together when vulcanizing, taken apart when melting wax, &c. The chimney is two inches in diameter at the place of attachment at the lower part of the sheath. An elbow extending back one and a half inches from the sheath carries up the tapering chimney fifteen inches, with the top three-quarters of an inch in diameter. A little glass window in front gives a good sight of the blaze.

The kerosene burns as free from smoke as in the ordinary glass chimneys. The heat is much easier controlled than with alcohol, and of course much cheaper.

